# Perceived Employability, Academic Commitment, and Competency of University Students During the COVID-19 Pandemic: An Exploratory Study of Student Well-Being

**DOI:** 10.3389/fpsyg.2021.788387

**Published:** 2021-12-17

**Authors:** Vincenza Capone, Leda Marino, Miriam Sang-Ah Park

**Affiliations:** ^1^Department of Humanities, University of Naples “Federico II”, Naples, Italy; ^2^Department of Psychology, Nottingham Trent University, Nottingham, United Kingdom

**Keywords:** perceived employability, academic commitment, university reputation, undergraduate students, COVID-19, mental well-being

## Abstract

Coronavirus disease 2019 (COVID-19) outbreak has led to the closure of schools and universities, which forced students to reorganize their daily and academic lives. The pandemic has thus impacted the well-being of students in various ways. This study aimed to investigate the relationship between the perceived employability, self-efficacy, ambition, organizational commitment, and career planning of students, as well as mental well-being, student engagement, and academic burnout during the pandemic. A total of 269 Italian university students participated in an online questionnaire. Our results highlight that students experienced high levels of uncertainty about their employability and career planning. In contrast, however, they reported healthy levels of mental well-being and student engagement, high career ambitions, and strong self-efficacy, despite the impact of COVID-19. We suggested that intervention and supportive programs should be offered to students over the long term in order to minimize the negative impact of the pandemic.

## Introduction

The McKinsey Global Institute (MGI) estimated that the unemployment rate in Europe could almost double in the next months as a result of the pandemic (Chinn et al., 2020). In Italy, the impact of coronavirus disease 2019 (COVID-19) is felt on work and employment: The number of employees in Italy fell by 444,000 units in 2020. Istituto nazionale di Statistica (2021) reported that the unemployment rate has risen to 9.0% (+0.2 points) in December 2020. Among young people, the situation is much worse, with the rate having increased to 29.7% (+0.3 points). Akkermans et al. (2020) argued that the pandemic will bring a career shock for many people. However, highlighting the importance of the dynamic interplay between individual and contextual factors, they also claim that some psychosocial resources make this career shock more manageable. Given the times, it is urgently needed that we explore the shifts in the sense of identity of young people and their career aspirations, which may be significantly affected by the crisis (Parola, 2020). Research on the perceptions of employability and well-being of young people is particularly relevant, as well as the ways to facilitate their transition into work, considering that they were already a vulnerable group before the crisis (Autin et al., 2020). Although it is known that high-quality education is consistently associated with career success, less is known of how the association works or how education prepares students for their future careers (Hogan et al., 2013). A big task for educators is thus to explore whether academic skills are transferable to employment settings (Hager and Holland, 2007). Graduates are becoming increasingly conscious that academic qualification alone may not be enough for getting a suitable job (Tomlinson, 2012) and that to achieve early career success in this turbulent labor market, they need to develop career competencies (Akkermans et al., 2013; Lo Presti et al., 2021). This had been supported by Bustamam et al. (2015) who highlighted that the importance of equipping graduates with career skills relevant to the market which they believe will give them better confidence and independence.

Mental well-being has become a profound issue during the pandemic, with studies on COVID-19 and mental health showing the negative psychological effect to be moderate to severe. About one-third of the population reported moderate-to-severe anxiety (Wang et al., 2020), and young people were identified as the highest-risk group for a range of mental illnesses (Huang and Zhao, 2020; Liang et al., 2020). In the higher education context, mental well-being has been associated with educational aspirations, academic engagement, academic achievement, and dropout (Marino and Capone, 2021). In young people, the well-being grows thanks to positive experiences with peers and significant adults in different settings, and universities can play a central role in the lives of students in the sense that they provide the opportunity to fulfill such needs for belonging and acceptance (Capone et al., 2020). Therefore, the well-being and the academic skill developments of students go hand in hand, with the university experience of students determining the outcomes for both (Lodi et al., 2021). However, the pandemic has had a big impact on the academic life of students, changing practices regarding social life (no meetings with friends, university colleagues, or teachers, no traveling), and the ways to live in the academic community changed drastically, with many restrictions imposed (Procentese et al., 2020). In-person teaching and campus activities were largely suspended with online activities, replacing all of the more traditional ways of interaction.

In the pandemic, some students have experienced stress due to the potential drop in the quality or satisfaction with the mode of teaching, finances, and other factors, which in turn could have affected their academic performance as well as mental well-being. It may thus be important to examine the levels of burnout and risk perception of students as well as their perceptions of university reputation as a way of gauging their mental well-being and career competencies in times of the pandemic. Schaufeli et al. (2002b) defined burnout among students as feeling tired due to the demands of study, having a cynical attitude to what was learned and feeling inept to complete the study. Burnout is thus assumed to affect academic performance and well-being, as it renders the sense of inadequacy and incompetence of a student (Schaufeli and Greenglass, 2001). In a related vein, risk perception is also one of the most relevant variables in determining the responses of individuals to global pandemic disease (Brug et al., 2009), in the sense that it can influence protective behaviors and other behavioral and emotional responses to the pandemic (Capone et al., 2020). If students perceive high risk in their academic and future careers as the result of the pandemic, they may be less motivated to work and also have reduced overall well-being. The demand for individuals with degrees in particular subjects varies not only according to market conditions but also depending on the perceived status differences between vocational areas. The brand image of university or its perceived reputation might influence the academic satisfaction of students as well as perceived employability. Bainbridge (2000) found that the “brand” of an existing employer on a resume lent credibility to the job-seeking potential of individuals. University reputation may build credibility and trust for its graduates and may increase the chances of their successfully getting a job (Bano and Vasantha, 2019).

Work engagement as a positive and fulfilling work mindset is characterized by vigor, dedication, and absorption with which employees perform work (Schaufeli et al., 2002b). This construct has been extended to evaluate the performance and engagement of students as an essential condition of a successful adaptation to the academic environment (Schaufeli et al., 2002a). The reasoning is that the activities students perform can also be considered as “work,” in the sense of goal-directed and structured activities, which are compulsory in nature (Carmona-Halty et al., 2019). The relationship between student engagement and well-being is still not well-understood, but some studies indicated a positive interaction between student, engagement, and happiness (Boulton et al., 2019). Work engagement has been found to be associated with different indicators of well-being such as personal accomplishment, health, self-efficacy, job satisfaction, and turnover intention (Schaufeli and Bakker, 2003; Schaufeli et al., 2006). The link between organizational commitment and well-being, for instance, has been well-explored, with the affective organizational commitment shown to be positively related to well-being (Panaccio and Vandenberghe, 2009). More recent studies (e.g., Atitsogbe et al., 2019; Lo Presti et al., 2021) have also consistently shown that higher perceived employability levels are linked to job-related and mental well-being, as well as career success.

The Conservation of Resources Theory (Hobfoll, 1989) conceived employability as a personal resource that correlates favorably to a wide array of both work-related and general well-being outcomes. Employability comprises human capital, social capital, personal characteristics, and personal behaviors that make graduates more likely to gain employment (Clarke, 2018). It appears to be a key quality for graduate students (Lo Presti et al., 2018), associated with competency, which in turn increases the likelihood of getting and maintaining a job (De Cuyper et al., 2012). As employability reflects the beliefs of individuals about their possibilities of getting new employment, it is connected to self-efficacy. Blokker et al. (2019), in a sample of young professionals, showed that those who developed their career competencies reported higher levels of subjective career success. More specifically, higher levels of career self-efficacy have been shown to be positively related to job search behavior and employment outcomes among recent graduates (Pinquart et al., 2003), and career planning and career insight were positively associated with subjective career success (Kuijpers et al., 2006). In the social-cognitive perspective (Bandura, 1997), self-efficacy is defined as the “belief in one's capabilities to organize and execute the courses of action required to produce given attainments” (Bandura, 1997, p. 3). Being confident in their abilities seems to play a key role in sustaining the employability of individuals and, consequently, enhances the overall chances of finding employment (Shumilova and Cai, 2015). Overall, the links between academic self-efficacy, student engagement, and mental well-being are well-established (Sovet et al., 2014; Marino and Capone, 2021), and there is also evidence for the relationship between self-efficacy and employability among graduates (Lo Presti et al., 2021). Moreover, self-efficacy has been shown to be positively related to the job search behavior and the subsequent employment outcomes of students (Pinquart et al., 2003). Higher levels of self-efficacy are also associated with lower levels of stress, anxiety, and depression (Bandura et al., 2001). The period after the first lockdown was full of uncertainties regarding the evolution of COVID-19 cases. This also brought uncertainty regarding how the universities shall proceed. Our research has focused on the relationship between student engagement and the mental well-being of students and their perceived employability, considering some variables related to the career competencies of students, such as career planning, organizational commitment, perceived university reputation, ambition, and academic self-efficacy. We also wanted to explore the relationships between all these variables and the risk perception and fear of COVID-19. We hypothesized that as follows:

H1: Perceived employability, mental well-being, and student engagement will be positively associated with one another;
H2: Perceived employability will be positively associated with the variables related to the career competencies of students: academic self-efficacy, career planning, ambition, organizational commitment, and perceived university reputation;
H3: Perceived employability will be negatively associated with the academic burnout of students, the risk perception to COVID-19, and the fear of contracting the virus.

## Materials and Methods

### Participants

The sample consisted of 269 Italian university students from the Federico II University of Naples, a large university in southern Italy. Participants ranged in age from 18 to 45 years (*M* = 22.99, SD = 2.78), and 77.3% of the sample was women. Of the 269 students, 68.8% were at the level of bachelor's degree, and 28.2% were doing their magistral degree. Further details on the demographic information of participants such as the type of course (e.g., humanities or (Science, Technology, Engineering and Mathematic) STEM subjects) can be found in Table 1.

**Table 1:** Sociodemographic characteristics of students (*N* = 269).

	***M***	***SD***
**Gender (%)**		
Male		
22.7		
Female		
77.3		
**Age range**		
18–45	22.99	2.78
**Bachelor's degree (%)**		
68.8		
Humanities studies (%)		
84.5		
STEM studies (%)		
15.5		
**Magistral degrees (%)**		
28.2		
Humanities study (%)		
36.4		
STEM studies (%)		
63.6		
**Exams completed**	14.9	7.1
**Average of marks**	27.5	2.7
**Students employed (%)**		
21.2		
**Type of workers (%)**		
Full-time		
23.4		
Part-time		
34.4		
Freelancer		
25		
Volunteer		
17.2		
**Working hours per week**	19.74	14.12

### Procedure

A cross-sectional study was performed during the second phase of the lockdown in Italy (May 2020). The students voluntarily completed an online self-report questionnaire on the Google Forms platform. Participation was anonymous, and no incentive was given. All participants were given the option to withdraw at any moment. The study was advertised in academic courses at the institute of the first author and on relevant pages/groups on social media platforms such as Facebook and Instagram. Data were collected through convenience sampling. We obtained approval from the Ethics Committee at the Department of Humanities, University of Naples, Federico II (Italy).

### Instruments

#### Sociodemographic Section

The questionnaire included a sociodemographic section that included questions related to the academic career of students, i.e., type of course, the number of university exams completed, and the average of the marks obtained, in addition to age and gender. Also, it was asked whether the students worked and which type (i.e., volunteer, part-time, or freelancer), as well as how many hours per week they spent in work activities. All the rest of the variables in the study were measured using Likert scales. High scores correspond to high levels of the construct.

#### Employability, Ambition, Organizational Commitment, and University Reputation

Self-perceived employability was assessed by the Students' Perceived Employability Scale (SPES; Rothwell et al., 2008). The scale consists of 16 items evaluated with a 5-point Likert scale ranging from 1 (strongly disagree) to 5 (strongly agree). An example of these items is “I regard my academic work as a top priority.”

Career ambition was assessed by the Ambition subscale from the Self-perceived Employability Scale (Rothwell et al., 2008), consisting of 6 items that are rated on a 5-point Likert scale as 1 (completely disagree) and 5 (completely agree). An example of these items is “I have clear goals for what I want to achieve in life.”

Organizational commitment was detected by the University Commitment Scale (Rothwell et al., 2008). This tool consists of 8 items evaluated with a 5-point Likert scale ranging from 1 (strongly disagree) to 5 (strongly agree). An example of these items is “I am proud to tell others I am at this university” (Rothwell et al., 2008, p. 3–5).

Finally, one item was used to measure university reputation: “My university has an outstanding reputation in my field(s) of study,” which was evaluated with a 5-point Likert scale ranging from 1 (strongly disagree) to 5 (strongly agree).

#### Career Planning

Career planning was measured using the Career Planning Scale (Gould, 1979). This tool measures the level at which individuals identify their skills, values, and interests, seeking different work paths to establish effective career planning. The scale consists of 6 items, evaluated with a 5-point Likert scale ranging from 0 (completely disagree) to 4 (completely agree). An example of these items is “I know how to achieve my career goals.”

#### Academic Self-Efficacy

The academic self-efficacy beliefs of students were assessed by the Student Self-efficacy Scale (Capone and e Petrillo, 2017). The tool consists of 6 items measuring the beliefs of students in their ability about their role. Students rated, on a 7-point Likert scale, their agreement ranging from 1 (strongly disagree) to 7 (strongly agree). An example of these items is “I can effectively resolve the issues about my studies.”

#### Academic Burnout of Students

The academic burnout of students was detected by the Maslach Burnout Inventory-Study Survey (Schaufeli et al., 2002b; Italian version developed by Borgogni and Consiglio, 2005). In this study, we considered two aspects of burnout, namely, emotional exhaustion, described as the feeling of being emotionally drained by study, and cynicism, which is a pessimistic response to the study. Notably, 5 items are related to emotional exhaustion, and 4 items are related to cynicism. The range of the answer is calculated on a 7-point Likert scale ranging from 0 (never) to 7 (always). An example of these items is “I have become less enthusiastic about my studies.”

#### Risk Perception of COVID-19 and Fear

The perception of risk of participants to COVID-19 was detected by the Risk Perception of COVID Scale (Capone et al., 2021), consisting of 4 items evaluated with a 5-point Likert scale ranging from 1 (strongly worried) to 5 (not worried at all). An example of these items is “Are you worried about getting infected with COVID-19 yourself?”

We also included a single item, i.e., “How scared are you from the COVID-19 emergency?” (Savarese et al., 2020, p. 6), about the worry for COVID-19 in a range of answers from 1 (not at all worried) to 10 (very worried).

#### Student Engagement

The student engagement construct was detected by the Utrecht Study Engagement Scale (Balducci et al., 2010). The tool consists of 14 items evaluated with a 7-point Likert scale ranging from 1 (never) to 7 (always) and measuring student engagement. An example of these items is “My studies inspire my life.”

#### Mental Well-Being

The mental well-being was detected by the Italian Mental Health Continuum-Short Form (Petrillo et al., 2015). The tool consists of 14 items evaluated with a 6-point Likert scale ranging from 1 (never) to 6 (every day). An example of these items is “During the past month, how often did you feel happy?”

### Data Analysis

Descriptive statistics were used to summarize variables collected in the questionnaire and the main characteristics of the study population. According to Vaske et al. (2017), alpha values between 0.65 and 0.80 are considered “adequate” for scales adopted for research on human dimensions. Pearson's correlation was performed to analyze the association between variables. The data analysis was performed using IBM SPSS Statistics 26.0.

## Results

The descriptive statistics of the variables are reported in Table 2. Results showed that the perceptions of employability are characterized on average by generalized uncertainty: students do not believe that in the immediate future they will find a job and that their degree can be employable. The greatest concern relates to placement in the geographical area of their university: it is perceived as particularly at risk in terms of employability, but that university reputation is the important factor for employability. However, despite the challenges posed by the pandemic, the students did not lose their career ambitions. It appears that they want to prepare themselves for the future career they have aspired to before the pandemic, so they continue to focus on studying. Referring to career planning, students declare that they are uncertain about the steps to be implemented with respect to managing their careers. The students declared that they feel committed to the organization: a feeling of pride prevails toward belonging to it. As regards to the self-efficacy of students, the participants scored very high. This means that they feel that they have the skills to face the tasks required by the university course and to be able to effectively reach the set learning objectives. The levels of engagement are very positive: students declare that they have quite frequently experienced positive feelings toward the study and the learning methods proposed during the lockdown period. Students also reported sufficient levels of mental well-being. Participants reported low levels of academic burnout, but students seem to report exhaustion at the end of the day of attendance of courses (online) which was declared as moderately high. In general, students were concerned about the possibility of being at risk of contagion. Of note, 44% of respondents also affirmed that they were particularly frightened by the health emergency that the country is experiencing (scores > 5, ranging from 1 to 10). Higher levels of perceived risk emerge with respect to the health of their families and the inadequacy of the protection measures implemented in Phase 2.

**Table 2:** Descriptive statistics (*N* = 269).

	***M***	***SD***	**Range of scale**	**Cronbach's alpha**
Employability	3.20	0.50	1–5	0.81
University reputation	3.60	0.87	1–5	//
Ambition	3.91	0.57	1–5	0.80
Career planning	2.24	0.83	0–4	0.81
Organizational commitment	3.23	0.66	1–5	0.84
Academic self-efficacy	5.46	0.96	1–7	0.86
Student engagement	3.12	1.11	0–6	0.92
Mental well-being	3.43	0.97	1–6	0.91
Students' academic burnout	1.83	1.16	0–6	0.89
Risk Perception of COVID-19	2.42	0.85	1–5	0.81
Fear to COVID-19	5.48	2.23	1–10	//

### Correlations

Zero-order correlations between the measures are shown in Table 3. Results indicate that mental well-being is negatively correlated with the academic burnout of students, while it is positively correlated with employability, university reputation, ambition, career planning, academic self-efficacy, and organizational commitment. Employability is positively correlated with university reputation, career planning, organizational commitment, academic self-efficacy, and student engagement. University reputation is positively correlated with ambition, organizational commitment, academic self-efficacy, and student engagement. Career planning is positively correlated with ambition and organizational commitment. Ambition is positively correlated with organizational commitment, academic self-efficacy, and student engagement. Organizational commitment is positively correlated with academic self-efficacy and student engagement. Academic self-efficacy is positively correlated with student engagement. Related to the perception of risk to COVID-19, it is negatively correlated with academic self-efficacy and positively correlated with fear to COVID-19, while the fear to COVID-19 has a low positive correlation with university reputation. Finally, the academic burnout of students is negatively correlated with employability, ambition, organizational commitment, academic self-efficacy, and student engagement. It is important to highlight a moderately high correlation between employability and mental well-being and the protective role of ambition toward the academic burnout of students.

**Table 3:** Correlations between variables (*N* = 269).

**Variables**	**1**	**2**	**3**	**4**	**5**	**6**	**7**	**8**	**9**	**10**	**11**
1. Employability											
2. University reputation	0.502^**^										
3. Ambition	0.273^**^	0.274^**^									
4. Career planning	0.265^**^	0.094	0.128^*^								
5. Organizational commitment	0.362^**^	0.342^**^	0.201^**^	0.223^**^							
6. Academic self-efficacy	0.188^**^	0.234^**^	0.467^**^	0.055	0.217^**^						
7. Student engagement	0.195^**^	0.120^*^	0.358^**^	0.077	0.323^**^	0.556^**^					
8. Mental well-being	0.421^**^	0.167^**^	0.278^**^	0.326^**^	0.285^**^	0.283^**^	0.321^**^				
9. Students' academic burnout	−0.154^*^	**−0.030**	−0.284^**^	0.025	−0.229^**^	−0.345^**^	−0.310^**^	−0.295^**^			
10. Risk perception of COVID−19	−0.033	0.004	−0.103	−0.012	0.011	−0.144^*^	−0.055	−0.002	−0.081		
11. Fear of COVID−19	0.033	0.134^*^	−0.070	−0.027	0.007	0.036	−0.069	−0.063	0.080	0.369^**^	

* *p < 0.05;*

** *p < 0.0001*.

*The bold values provided in table are the most significant correlations to explain our results*.

## Discussion

This study specifically focused on Italian university students, examining the relationship between employability and career competencies alongside other psychological outcomes during Phase 2 of the pandemic (immediately after the first lockdown). We argued that career competencies can support young individuals in facing the pandemic and university life. Our findings mostly supported the hypotheses. There were significant and positive associations between career competencies, student engagement, and mental well-being, and negative associations with burnout. The threats posed by COVID-19 have had an impact on the academic community and university students. During the pandemic, students' professional placements and internships were interrupted, forcing them to continue these activities in online mode. This indicates that more scholarly, along with policy level and educational level, attention is needed to better understand and, as a result, increase the chances of the entry of graduates into the labor market and early career success (Capone et al., 2020; Lo Presti et al., 2021). One of the challenges that the present universities face is to ensure that their students serenely continue their studies and find employment in line with their aspirations.

This study focused on the competencies and well-being of students during forced adaptation to the full-online education amidst other challenges of the pandemic restriction, the psychological impact related to the physical distancing protective measures, and their employability. Evidence is promising and sheds light on an important phenomenon, having both theoretical and applied implications. To our knowledge, this is the first study to investigate the impact of COVID-19 on student engagement and mental well-being of Italian university students during the lockdown. Surprisingly, we did not find a significant relationship between fear of COVID-19, risk perception, academic variables, and well-being. While expressing some concerns regarding the pandemic, the well-being and career competencies of students did not seem much affected. Variables, such as technostress and lack of relationships with colleagues and teachers, could mediate the relationship between these variables. More studies are needed to further explore these relationships and, more importantly, identify the protective factors.

From a practical point of view, tailored academic interventions could be implemented to target either separately or jointly the self-efficacy, career planning, and employability of students, as well as to foster specific aspects of well-being. Intervention and supportive programs based on improving these variables, as well as confidence in relationships, should be offered to students over the long term, beyond the current outbreak. Our research also sheds a light on the importance of strengthening the affective commitment of students and the ways of managing university reputation. Affective commitment satisfies the basic psychological needs of employees and provides positive emotions, which, in turn, help restore limited regulatory resources (Rivkin et al., 2015). Thus, affective commitment should buffer the negative psychological effect of the pandemic. The correct management of reputation and image can be crucial to guarantee the survival and success of organizations (Del-Castillo-Feito et al., 2019). Higher education institutions have understood the importance of managing intangible assets such as image to differentiate themselves over competitors and to improve the relationship with their stakeholders (Nguyen and LeBlanc, 2001). Our study goes in the direction of promoting reputation also to favor the career choices and well-being of students, especially in a period of uncertainty. Results should be deepened in subsequent phases of the pandemic. Universities can offer access to support and therapy and thus reduce short- and long-term impact of the crisis.

Previous studies have demonstrated that how young people, in particular university students, are at greater risk of being discouraged about the future and career choices in case of a health emergency (Aristovnik et al., 2020). Uncertainty for the future and anxiety are core feelings for both in this pandemic. Governments and organizations (such as universities) should begin thinking about how to deal with the immediate and long-term consequences of the economic crisis created by COVID-19, especially in the area of employment. It requires individuals to self-manage their careers, bearing the brunt of the responsibility for their own employability by relying on key factors, such as proactivity, adaptability, and entrepreneurial thinking and acting (Obschonka and Stuetzer, 2017; Donald et al., 2018; Römgens et al., 2020). The recommendation was for universities to encourage more employer involvement in the selection of employability criteria and for greater employment-based training and experience (Mason et al., 2009). While further research is needed for more detailed evaluation, our finding may be in line with the study by Hager and Holland (2007), who recommended that academics need to redesign their curricula and introduce new methodologies to improve the employability of students. Researchers have acknowledged that employability is a disposition encompassing both reactive and proactive characteristics, meaning that employable individuals are both able to adapt reactively to known demands and to proactively create and realize opportunities (Fugate and Kinicki, 2008).

Despite the importance of our findings, some limitations should be noted. First of all, our study relied on a convenience sample of Italian university students, which limits the generalizability of the results. As such, further research should be designed to replicate this study to the representative samples. Furthermore, considering evidence regarding the type of degree course and varying the levels of well-being and perceived employability could be useful (Bison et al., 2019), especially in the COVID-19 situation in which different sectors are impacted to varying levels (Kamaruddin et al., 2021). The data we collected were at a single point in time, so it is a correlational study, while a longitudinal design could have allowed for examining causal links among variables. Finally, this study used self-report measures to collect the data, and further studies would benefit from assessing the constructs using multiple informants.

## Data Availability Statement

The raw data supporting the conclusions of this article will be made available by the authors, without undue reservation.

## Ethics Statement

The studies involving human participants were reviewed and approved by University of Naples Federico II. The patients/participants provided their written informed consent to participate in this study.

## Author Contributions

VC and LM contributed to conception and design of the study and performed the statistical analysis. LM organized the database. VC, LM, and MP wrote the first draft of the manuscript. All authors contributed to manuscript revision, read, and approved the submitted version.

## Conflict of Interest

The authors declare that the research was conducted in the absence of any commercial or financial relationships that could be construed as a potential conflict of interest.

## Publisher's Note

All claims expressed in this article are solely those of the authors and do not necessarily represent those of their affiliated organizations, or those of the publisher, the editors and the reviewers. Any product that may be evaluated in this article, or claim that may be made by its manufacturer, is not guaranteed or endorsed by the publisher.
